# Study on the diversity, structure, and function of endophytic bacteria in seeds of genuine medicinal plants in gansu province

**DOI:** 10.1186/s12870-026-08767-5

**Published:** 2026-04-18

**Authors:** Shanjia Li, James F White, Yixiao Zhai, Xinrong Li, Kerong Wang

**Affiliations:** 1 School of Life Science and Engineering, Lanzhou University of Technology, Lanzhou, Gansu 730000 China; 2 Department of Plant Biology, Rutgers University, New Brunswick, NJ 08901 USA; 3 Key Laboratory of Land Surface Process and Climate Change in Cold and Arid Regions, Northwest Institute of Eco-Environment and Resources, Chinese Academy of Sciences, Lanzhou, 730000 China

**Keywords:** Medicinal plants, Seed microbiome, Plant growth-promoting (PGP) bacteria, Core microbiome, Bacterial isolation

## Abstract

**Background:**

Seeds harbor diverse endophytic bacterial communities that are critical for seed health and plant fitness, however, their functional potential in medicinal plants remains underexplored. This study employed integrated high-throughput sequencing and culturomics to characterize the endophytic bacterial communities in seeds of 10 endemic medicinal plants from Gansu, China, and evaluate their roles in germination enhancement.

**Results:**

We identified 26,167 bacterial OTUs, with 19 core OTUs consistently present across all samples. The dominant phyla were Proteobacteria, Firmicutes, Bacteroidota, and Actinomycetota, collectively comprising over 80% of relative abundance. Plant species identity was the primary factor shaping bacterial community structure. Among 89 isolated endophytic bacterial strains, 72% exhibited multiple plant growth-promoting (PGP) traits in vitro, including indole-3-acetic acid (IAA) synthesis, siderophore production, phosphate solubilization, and nitrogen fixation. Preliminary validation experiments using wheat (*Triticum aestivum* ‘Jimai 22’) under controlled conditions indicated that seed-derived *Bacillus* and *Pseudomonas* strains significantly promote radicle elongation and improve vigor indices under saline-alkaline stress compared to axenic controls.

**Conclusions:**

These findings highlight medicinal plant seeds as reservoirs of culturable bacteria with multifunctional PGP capabilities. The host-specific community patterns and the in vitro PGP traits suggest these isolates warrant further investigation as potential candidates for bioinoculant development, pending validation on the host medicinal plants themselves.

**Supplementary Information:**

The online version contains supplementary material available at 10.1186/s12870-026-08767-5.

## Introduction

Plant endophytic bacteria, which inhabit plant tissues during specific or entire life stages [1], form complex symbiotic networks that profoundly influence host fitness [2, 3]. These microbial partners exhibit remarkable diversity across plant species, organs, genotypes, and edaphic conditions [4] and are acquired through vertical transmission from parental plants or horizontal recruitment from environmental reservoirs [5]. As critical reproductive units, seeds represent unique ecological niches that preserve vertically transmitted bacterial communities, which collectively orchestrate essential biological processes from enhancing germination efficiency to programming subsequent plant-microbe interactions [6–9]. Mounting evidence reveals seed-borne bacteria confer multifaceted benefits, including phytohormone synthesis (e.g., IAA) [10], nutrient acquisition (nitrogen fixation [11], phosphate solubilization [12]), and pathogen suppression through siderophore-mediated competition [13, 14], with their depletion causing significant host fitness penalties [15].

The structural and functional dynamics of seed bacterial microbiomes are shaped by an evolutionary interplay between host selection [16] and environmental filtering [17]. While conserved core taxa (e.g., *Bacillus*, *Pseudomonas*) dominate across plant species [18, 19], community composition exhibits host-specific patterns influenced by domestication history and agricultural practices [20]. Crucially, these bacterial assemblages serve dual roles as both plant health determinants [21] and potential pathogen vectors [22], with seed-transmitted pathogens like *Fusarium* posing global biosecurity risks. This functional dichotomy underscores the urgency to decipher the assembly rules governing seed bacterial microbiome formation [23] and transmission-particularly [24] in medicinal plants where seed quality directly impacts therapeutic compound biosynthesis [25].

In Gansu Province, China’s traditional herbal medicine cultivation hub [26], intensive farming practices threaten the sustainability of characteristic medicinal species (*Angelica sinensis* (Oliv.) Diels, *Codonopsis pilosula* (Franch.) Nannf., *Astragalus membranaceus* (Fisch.) Bunge) [27]. Excessive agrochemical use has triggered soil degradation and phytochemical profile alterations, necessitating microbiome-based solutions to restore plant health and medicinal quality. Although rhizosphere microbiomes have been investigated for crop protection, the functional potential of seed endophytic bacteria in medicinal species remains underexplored. Current understanding suffers critical gaps: (1) limited knowledge of vertically transmitted core bacterial microbiota across key medicinal taxa; (2) unresolved relationships between seed endophytic bacterial communities and host plant traits; and (3) insufficient characterization of culturable beneficial bacterial for agricultural applications.

To address these knowledge gaps, we conducted integrated 16 S rRNA gene amplicon sequencing and culture-based analyses of 10 characteristic medicinal species from Gansu’s Dao-di production regions. We characterized the diversity, structure, and putative functions of seed endophytic bacterial communities and established a culture collection for functional screening. Specifically, we aimed to: (1) Decipher host-specific patterns in seed endophytic bacterial microbiome assembly among Dao-di medicinal plants; (2) Identify core and culturable bacterial taxa with in vitro PGP and other traits; (3) Preliminarily evaluate the in vitro PGP potential and stress mitigation effects of selected isolates on a model plant under controlled conditions. Our study provides a systematic profile of seed endophytic bacterial biodiversity in Northwest China’s medicinal flora, offering foundational data and microbial resources for future research on seed bank management and sustainable cultivation.

## Materials and methods

### Sample collection

Seed samples of 10 traditional medicinal plants from Gansu Province were collected in Wuwei City, Gansu Province (37°93’ N, 102°64’ E). The species included were: *Astragalus membranaceus* (Fisch.) Bunge (AM), *Hedysarum polybotrys* Hand.-Mazz. (HP), *Glycyrrhiza glabra* L. (GG), *Glycyrrhiza inflata* Batalin (GI), *Glycyrrhiza uralensis* Fisch. (GU), *Glycyrrhiza pallidiflora* Maxim. (GP), *Hansenia weberbaueriana* Pimenov & Kljuykov (NI), *Bupleuri Radix* (RB), *Codonopsis pilosula* (Franch.) Nannf. (CP), and *Scutellaria baicalensis* Georgi (SB). Full Latin names are provided here upon first mention; abbreviations (in parentheses) are used consistently throughout the manuscript and figures. Mature, healthy seeds were collected using sterilized tools, placed in sterile sampling bags, and transported to the laboratory on ice. Seeds were stored at 4 °C until processing. All plant materials were identified by Professor Li Shanjia of Lanzhou University of Technology. These seed materials are preserved in the laboratory. Voucher specimens were not deposited in a public herbarium.

### Seed surface sterilization

For each of the 10 plant species, 3 g of seeds (biological triplicates) were surface-sterilized. Seeds were rinsed three times with sterile distilled water. Subsequently, they were agitated in 4% (v/v) sodium hypochlorite (NaClO) for 3 min [28], followed by immersion in 70% (v/v) ethanol for 5 min. Seeds were then washed five times with sterile distilled water. To verify the effectiveness of surface sterilization, 100 µL of the final rinse water was spread onto Luria-Bertani (LB) agar plates and incubated at 28 °C for 72 h; only batches with no microbial growth were used for downstream analysis. The sterilized seeds were divided: one portion was flash-frozen in liquid nitrogen and stored at -80 °C for DNA extraction, and the other was used immediately for the isolation of culturable endophytic bacteria.

### DNA extraction, amplification, and sequencing

Surface-sterilized, frozen seeds were freeze-dried and ground to a fine powder in liquid nitrogen. Total genomic DNA was extracted from approximately 1 g of powdered seed material per replicate using the DNeasy PowerSoil Pro Kit (Qiagen, Germany) according to the manufacturer’s instructions. DNA concentration and purity were assessed using a NanoDrop One spectrophotometer (Thermo Fisher Scientific, USA). The V3–V4 hypervariable regions of the bacterial 16 S rRNA gene were amplified using the primers 338 F (5′-ACTCCTACGGGAGGCAGCAG-3′) and 806R (5′-GGACTACHVGGGTWTCTAAT-3′) [29]. PCR amplification, purification, and library construction were performed following standard protocols. The final libraries were sequenced on an Illumina NovaSeq 6000 platform (Novogene, Beijing, China) using a 2 × 250 bp paired-end strategy.

### Isolation and screening of culturable endophytic bacteria

To comprehensively isolate culturable endophytic bacteria from seeds of all 10 medicinal plant species, we employed seven different culture media: Potato Dextrose Agar (PDA), Nutrient Agar (NA), LB, Tryptic Soy Agar (TSA), Reasoner’s 2 A Agar (R2A), Oatmeal Agar (OA), and Gauze’s No. 1 Synthetic Agar. All seven media were used for each plant species. For each species, surface-sterilized seeds were processed using two methods on all media to maximize recovery: (1) Direct Plating: Six intact seeds were placed on each medium plate supplemented with sterile water (Figure S3). (2) Homogenate Plating: Remaining seeds were homogenized in sterile saline (0.85% NaCl), serially diluted (10⁰ to 10⁻²), and 100 µL of each dilution was spread onto the media plates. All plates were incubated at 28 °C in the dark for 2–7 days and monitored daily for colony emergence. For each plant species, colonies exhibiting distinct morphologies (size, shape, color, margin) emerging from both plating methods across all media types were selected as representatives of the cultivable diversity. These colonies were purified by repeated streaking on their respective isolation media until axenic cultures were obtained (Figure S4-S12). Purified strains were cultured in nutrient broth and preserved at -80 °C in broth containing 50% (v/v) glycerol for long-term storage.

### Strain identification

Genomic DNA from pure bacterial cultures was extracted using a bacterial DNA kit (TaKaRa, Japan). The near-full-length 16 S rRNA gene was amplified using the universal primers 27 F (5′-AGAGTTTGATCCTGGCTCAG-3′) and 1492R (5′-GGTTACCTTGTTACGACTT-3′) [30]. PCR products were purified and sequenced commercially (Genewiz, Suzhou, China). The obtained sequences were assembled and compared against the EzBioCloud database for taxonomic identification. The raw sequencing reads generated in this study have been deposited in the Zenodo repository and are publicly available under the accession DOI: 10.5281/zenodo.15855276.

### In vitro plant growth-promoting (PGP) trait assays

All PGP trait assays were performed in triplicate.

IAA production was quantified as described by Gordon and Weber [31] with modifications. Bacteria were grown in LB broth supplemented with 1 mg/mL L-tryptophan for 5 days. The culture supernatant was mixed with Salkowski’s reagent, and IAA concentration was determined spectrophotometrically at 530 nm using a standard curve.

Siderophore production was detected on Chrome Azurol S (CAS) agar plates as per Schwyn and Neilands [32]; orange halos around colonies after 7 days indicated positive results.

Phosphate solubilization was assessed on National Botanical Research Institute’s Phosphate (NBRIP) agar [33]; clear halos around colonies after 7 days indicated solubilization, and the solubilization efficiency (halo diameter/colony diameter) was calculated.

Nitrogen fixation potential was screened by growth on Ashby’s nitrogen-free mannitol agar after 7 days of incubation at 28 °C [34].

### Bacterial suspension preparation for plant assays

Six bacterial strains—AM-5, AM-8, NI-1, NI-4, RB-9, and RB-12 (detailed characteristics are provided in Supplementary Tables S1–S9)—selected based on their strong in vitro PGP profiles, were used. Each strain was cultured in LB broth at 28 °C with shaking (180 rpm) until the late-exponential phase (OD_600_ = 1.0). Cells were harvested by centrifugation (5,000 × g, 5 min), washed twice, and resuspended in sterile distilled water, 150 mM NaCl solution, or 100 mM NaHCO₃ solution to a final OD_600_ of 1.0. Representative images of bacterial cultures are provided in Supplementary Figs. S3–S12.

### Wheat seed treatment and growth-promotion assay

Wheat (*Triticum aestivum* cv. ‘Jimai 22’) was chosen as a model plant for the initial functional validation experiments. This choice was based on its rapid, uniform, and well-characterized germination, standardized protocols for assessing salt-alkali stress responses, and its widespread use as a model system for screening PGP bacteria. This approach allowed for a controlled, efficient, and comparative assessment of bacterial effects before future, more complex studies on the host medicinal plants themselves. Wheat seeds were surface-sterilized (70% ethanol for 1 min, 4% NaClO for 3 min, followed by thorough rinsing). Sterilized seeds were soaked in the prepared bacterial suspensions (or corresponding sterile water/stress solution controls) for 5 h. For germination assays, seeds were placed on sterile moist filter paper in Petri dishes. For seedling growth assays, germinated seeds were transplanted into pots (5 seedlings per pot) containing an autoclaved mixture of peat and vermiculite (1:1, v/v). The potting mixture was pre-moistened with the respective treatment solution: distilled water (control), 150 mM NaCl (salt stress), or 100 mM NaHCO₃ (alkali stress). The experiment followed a completely randomized design with three replicate pots per treatment combination (bacterial strain × stress condition). Plants were grown in a controlled climate chamber. Bacterial suspensions (or control solutions) were applied as a soil drench (50 mL per pot) once a week for four weeks. After 30 days, plants were harvested for analysis of growth parameters and physiological indices.

### Physiological and biochemical analyses

Chlorophyll content was measured spectrophotometrically after extraction of leaf tissue in 95% ethanol, and concentrations were calculated using standard formulas [35]. Malondialdehyde (MDA) content, an indicator of lipid peroxidation, was determined using the thiobarbituric acid (TBA) method [36]. Free proline content was quantified using the acid ninhydrin method [37]. The activities of the antioxidant enzymes peroxidase (POD), superoxide dismutase (SOD), and catalase (CAT) were measured using specific commercial assay kits (Solarbio, Beijing, China) according to the manufacturer’s instructions.

### Statistical analysis

Raw 16 S rRNA gene sequencing reads were processed using QIIME2 (version 2020.6). Primers were trimmed using cutadapt. Denoising, chimera removal, and amplicon sequence variant (ASV) generation were performed using the DADA2 plugin. Taxonomic assignment of ASVs was carried out using the classify-sklearn naive Bayes classifier against the SILVA 138 database (for bacteria). Alpha diversity indices (Observed ASVs, Shannon, Simpson, Chao1, ACE) were calculated. Beta diversity was assessed using Bray-Curtis distances and visualized via Principal Coordinates Analysis (PCoA) and Non-metric Multidimensional Scaling (NMDS). Permutational multivariate analysis of variance (PERMANOVA, 999 permutations) was used to test for significant differences in community composition. Differential abundance analysis of taxa between groups was performed using the DESeq2 package in R. Putative metabolic functions of the endophytic bacterial communities were predicted using both the PICRUSt2 pipeline (mapped to KEGG pathways) and the FAPROTAX database (version 1.2.4) to specifically highlight biogeochemical cycles, including nitrogen metabolism. All statistical analyses and visualizations were performed in R (version 4.0.4) using packages including ‘phyloseq’, ‘vegan’, ‘ggplot2’, ‘DESeq2’, and ‘pheatmap’. For plant assay data, one-way or two-way analysis of variance (ANOVA) was performed, followed by Fisher’s Least Significant Difference (LSD) post-hoc test (*p* < 0.05) using SPSS software (version 26.0). Data are presented as mean ± standard error (SE).

## Results

### Composition and diversity of seed endophytic bacterial communities

**Fig. 1:**
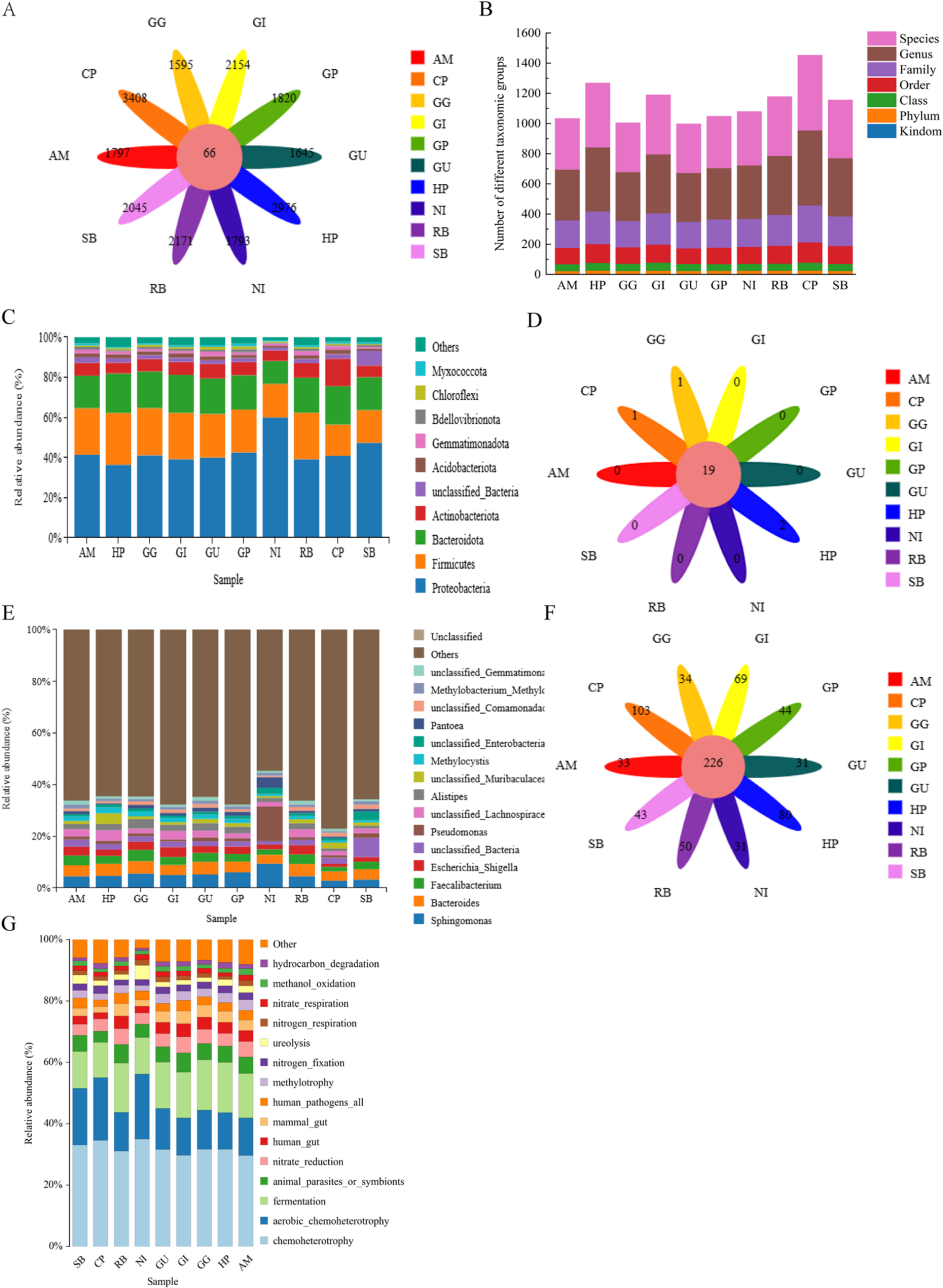
Composition, diversity, and predicted functions of seed endophytic bacterial communities in ten medicinal plant species. **A** Venn diagram showing shared and unique OTUs among the ten seed microbiomes. **B** Taxonomic richness at phylum, class, order, family, and genus levels across different host species. **C** Relative abundance of the dominant bacterial phyla in each seed sample. **D** Relative abundance of the dominant bacterial genera in each seed sample. **E** Number of shared phyla and genera among all ten seed microbiomes. **F** Number of unique phyla and genera identified in each host species. **G** Functional prediction of endophytic microbial communities in different seeds based on FAPROTAX

High-throughput sequencing of the bacterial 16 S rRNA V3-V4 regions from seeds of 10 medicinal plants revealed diverse and host-specific endophytic bacterial communities. From 2,434,096 high-quality sequences, we identified 26,167 OTUs. A very small core microbiome of only 66 OTUs (0.25%) was shared across all plant species, underscoring high interspecific variability (Fig. 1A). Instead, the communities were dominated by species-specific OTUs (24,045 OTUs, 91.9%), with CP harboring the most unique OTUs (3,408).

Alpha diversity analysis showed no significant differences in community richness (Chao1, ACE) among hosts but revealed significant variation in diversity (Shannon, Simpson), with SB and NI displaying the highest and lowest diversity, respectively (Figures S1A-S1D). Beta diversity analysis (PCoA) confirmed that bacterial community structure was significantly influenced by host plant species (Figure S1E).

Taxonomically, we annotated sequences across 40 phyla and 1,315 genera. SB showed the highest taxonomic richness, while a GU1 showed the lowest (Fig. 1B). At the phylum level, four dominant phyla—Pseudomonadota, Bacillota, Bacteroidota, and Actinomycetota—.

consistently constituted over 82.58% of all communities, with Pseudomonadota being most abundant (Fig. 1C).

Genus-level analysis revealed 1,315 taxa, with 16 genera exceeding 1% relative abundance. The top five genera (*Sphingomonas*,* Bacteroides*,* Faecalibacterium*,* Escherichia*,* Pseudomonas*) exhibited significant interspecific disparities, as summarized in the differential abundance matrix. Specifically, leguminous seeds (AM, RB, GU2–4) were enriched in *Sphingomonas*, whereas *Bacteroides* dominated in CP, SB, and HP. Notably, NI uniquely harbored a high abundance of *Pseudomonas* with reduced community evenness. Other genera showed distinct patterns: for instance, *Faecalibacterium* was enriched in GG and GI, while Escherichia exhibited a positive enrichment in GP but a negative trend in HP (Fig. 1E).

Core microbiome analysis identified 19 phyla shared across all seeds, with species-specific phyla including Halanaerobiaeota and Hydrogenedentes in RB, Latescibacterota in CP, and Thermotogota in GU2 (Fig. 1D). At the genus level, 219 core genera coexisted across all species. However, unique genus assignment varied among hosts: CP contained the highest number of unique genera (92), followed by RB (72) and GU3 (62) (Fig. 1F).

### Functional prediction of seed endophytic bacterial communities

Functional profiling was performed using PICRUSt2 and the FAPROTAX database. PICRUSt2 analysis showed that metabolic pathways dominated the predicted functions (76.64–78.31%). Seeds of NI exhibited distinct functional potentials, with higher relative abundance in categories like Cellular Processes and Environmental Information Processing but lower in core metabolism and genetic information processing compared to others (Figure S2A). Species-specific functional specialization was evident at a finer resolution (Figure S2B).

To predict the metabolic potential of endophytic bacterial communities, we performed FAPROTAX functional annotation based on 16 S rRNA gene data. The functional categories were consistently predicted across all seed samples, including hydrocarbon degradation, methanol oxidation, nitrate respiration, nitrogen respiration, ureolysis, nitrogen fixation, methylotrophy, human pathogenicity, mammal gut association, human gut association, nitrate reduction, animal parasites/symbionts, and fermentation (Fig. 1G). Most functions showed high abundance in the first six hosts (SB, CP, RB, NI, GU, GG), indicating a core set of conserved metabolic capabilities. However, differential patterns were observed in the remaining three hosts (HP, AM, Other). Specifically, hydrocarbon degradation was negatively enriched in HP, while methanol oxidation exhibited a > 1‑fold increase in HP relative to other hosts. Nitrate and nitrogen respiration were lower in HP. Notably, human gut‑associated function displayed a unique profile: HP lacked this function, whereas AM retained it, suggesting host‑specific selection against certain gut‑related traits. Nitrogen fixation showed an ambiguous pattern in HP, warranting further validation. Fermentation data were incomplete and require re‑analysis.

### Isolation and identification of culturable seed endophytic bacteria

**Fig. 2:**
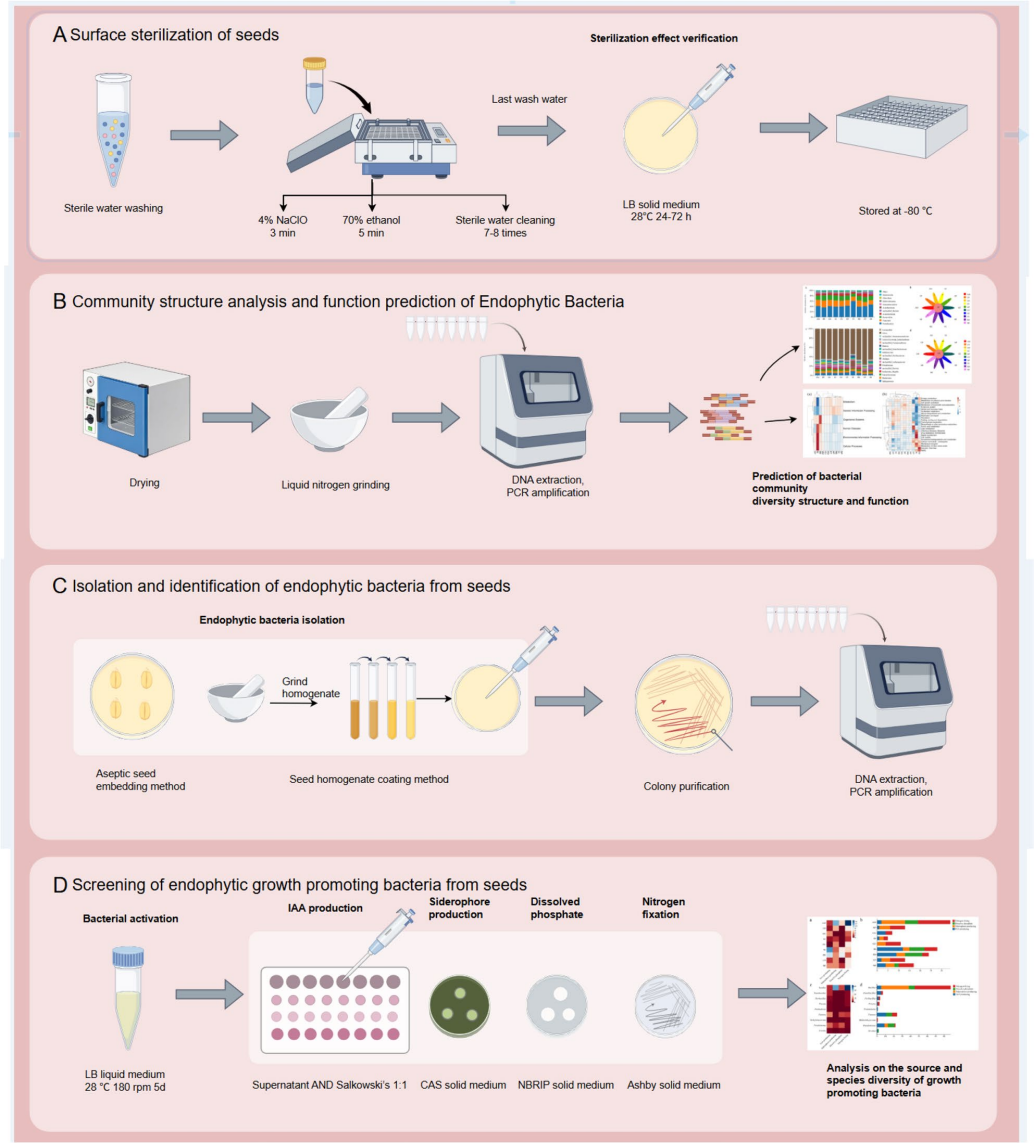
Experimental workflow for seed surface sterilization, microbial community analysis, bacterial isolation, and functional screening. **A** Schematic of the surface sterilization procedure and sterility verification. **B** Workflow for high-throughput 16 S rRNA gene amplicon sequencing, bioinformatic analysis, and functional prediction (PICRUSt2, FAPROTAX). **C** Isolation of culturable endophytic bacteria using seven different media and two plating methods (direct plating and homogenate plating), followed by purification and 16 S rRNA gene sequencing. **D** In vitro screening of isolates for PGP traits: IAA production, siderophore production, phosphate solubilization, and nitrogen fixation

**Fig. 3:**
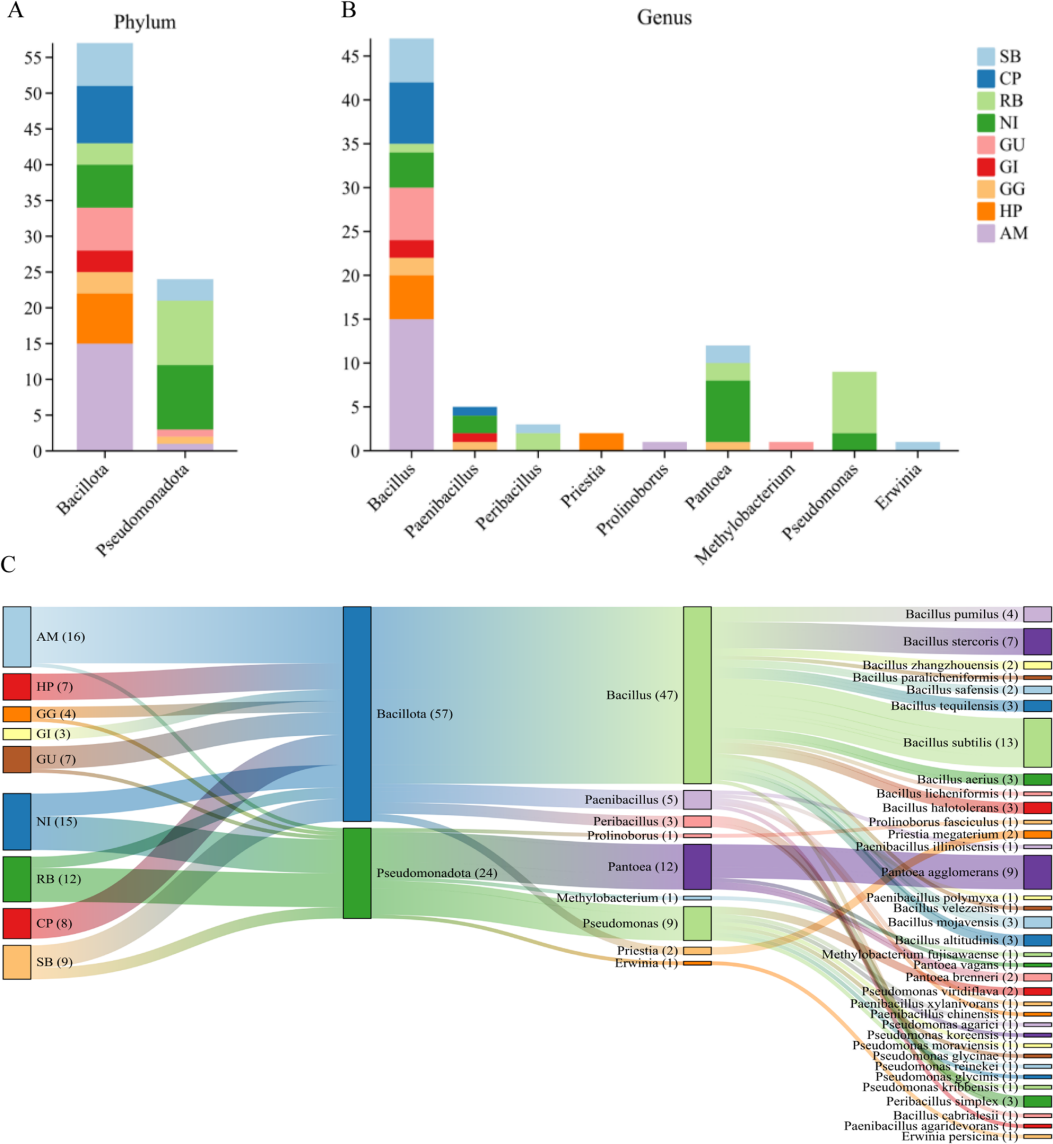
Taxonomic classification of culturable endophytic bacteria isolated from medicinal plant seeds. **A** Number of isolates assigned to bacterial phyla (*Firmicutes* and *Proteobacteria*). **B** Number of isolates assigned to bacterial genera; Bacillus was the most prevalent genus. **C** Species-level distribution of isolates across the nine host species from which bacteria were successfully cultured (no isolates were obtained from GP). Each colour represents a distinct bacterial species

Guided by the insights from the differential abundance and functional prediction analyses, which highlighted specific hosts and taxa as potential reservoirs for beneficial functions, we proceeded to isolate culturable endophytic bacteria from all 10 medicinal plant seeds using seven distinct culture media (Fig. 2). Despite multiple attempts, no endophytic bacteria were isolated from the seeds of GP. A total of 89 bacterial isolates, representing 37 species, were obtained. 16 S rRNA gene sequencing classified them into two phyla (*Bacillot*a and *Pseudomonadota*) and nine genera (Fig. 3A and B). *Bacillota* was dominant (65 strains), primarily represented by the genus *Bacillus* (55 strains), which aligned with its identification as a significantly enriched taxon in the differential analysis. *Pseudomonadota* accounted for 24 isolates.

Consistent with the sequencing-based findings, host-specificity was observed among the isolates. AM and HP yielded the highest cultivable species richness (10 each). *Bacillus* colonized seeds of all plant species. Notably, several unique genera were isolated exclusively from specific hosts, such as *Priestia* from RB and *Erwinia* from NI, mirroring the unique taxonomic signatures detected in the community sequencing data (Fig. 3C).

### PGP trait characterization of culturable endophytic bacteria

In vitro screening of the 89 isolates for four PGP traits, namely IAA production, siderophore production, phosphate solubilization, and nitrogen fixation, revealed significant functional variation. Isolates from *Glycyrrhiza uralensis* seeds exhibited only two PGP functions, whereas those from other plants possessed three or more.

**Fig. 4:**
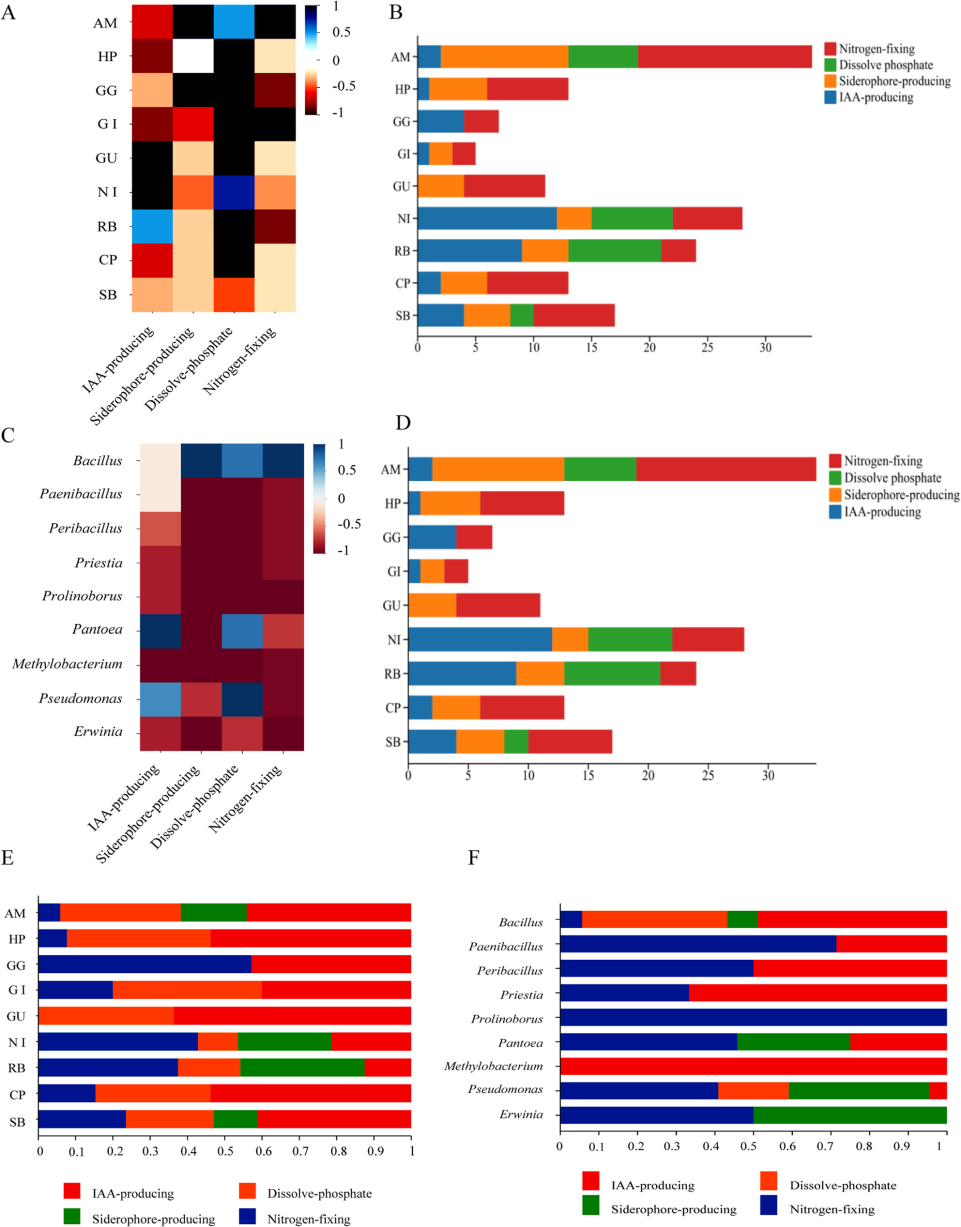
In vitro PGP traits of seed-endophytic bacterial isolates. **A** Heatmap showing the presence (coloured) or absence (grey) of four PGP traits (IAA production, siderophore production, phosphate solubilization, nitrogen fixation) among isolates grouped by host plant species. **B** Bar plot summarizing the number of isolates positive for each PGP trait in each host species. **C** Heatmap showing the distribution of the four PGP traits across different bacterial genera. **D** Bar plot summarizing the number of isolates positive for each PGP trait within each genus

IAA production was detected in 28 species, with RB and NI seeds contributing nine and seven IAA-producing species, respectively. No IAA-producing strains were isolated from GU. Siderophore production was observed in 18 species, predominantly from AM seeds (eight species). Phosphate solubilization occurred in 18 strains, mainly from RB (eight) and NI (six). All seed sources yielded nitrogen-fixing bacteria (23 species), with AM contributing ten species. AM, RB, and NI isolates displayed the most potent PGPR potential (Fig. 4A and B).

Genus-level analysis revealed functional specialization (Fig. 4C and D). IAA producers spanned eight genera, dominated by *Bacillus* and *Pantoea*. Siderophore producers clustered in *Bacillus* and *Pseudomonas*. Phosphate solubilizers were primarily *Pseudomonas* and.

*Bacillus*. Nitrogen fixers distributed across seven genera, with *Bacillus* predominating.

*Bacillus* encompassed all four PGP functions, while *Pseudomonas* exhibited multifunctionality.

### Effects of bacterial inoculation on wheat germination under saline-alkali stress

**Fig. 5:**
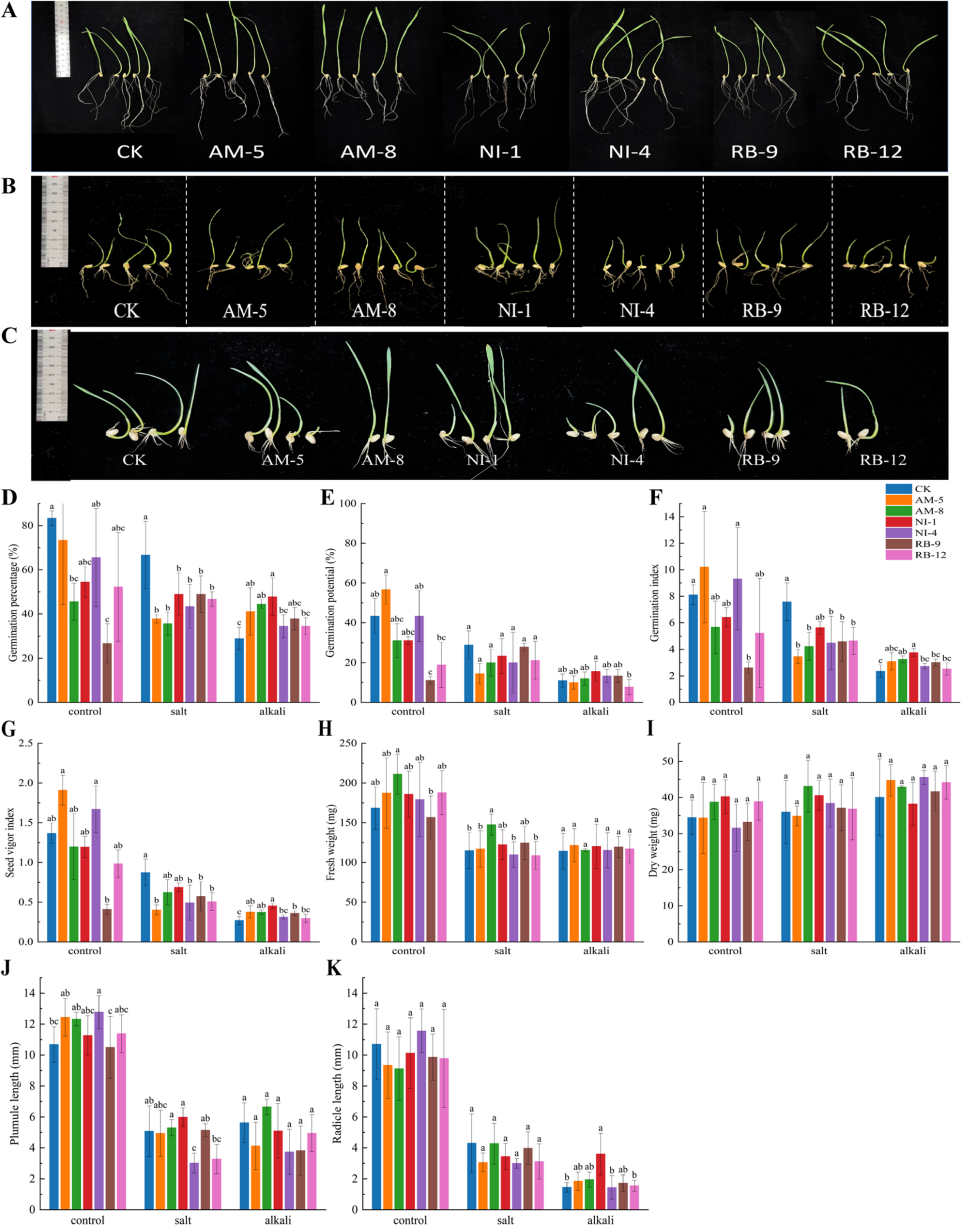
Effects of six selected endophytic bacterial strains on wheat seed germination under normal, salt, and alkali conditions. **A**–**C** Representative images of germinated seeds after 7 days under a normal (control), **B** salt stress (150 mM NaCl), and **C** alkali stress (100 mM NaHCO₃) with or without bacterial inoculation. **D** Germination rate, **E** germination potential, **F** germination index, **G** seed vigor index, **H** fresh weight, **I** dry weight, **J** coleoptile length, and **K** radicle length. Data are means ± SE (*n* = 3). Asterisks indicate significant differences compared to the respective non-inoculated control (*p < 0.05, **p < 0.01, ***p < 0.001)

The growth-promoting effects of six selected endophytic bacterial strains (AM-5, AM-8, NI-1, NI-4, RB-9, RB-12) were evaluated on wheat seed germination under normal, salt (150 mM NaCl), and alkali (100 mM NaHCO₃) conditions (Fig. 5A and C).

Under normal conditions, most strains slightly suppressed the germination rate. However, AM-5 increased germination potential by 30.78%, and both AM-5 and NI-4 significantly enhanced the germination index and vigor index significantly (Fig. 5D and G). Under salt stress, all strains inhibited germination rate and potential. Notably, AM-8 significantly increased seedling fresh weight by 28.30% and dry weight by 20.06% (Fig. 5H and I). Under the more severe alkali stress, NI-1 performed optimally, increasing germination rate, potential, and radicle length by 65.38%, 55.56%, and 148.28%, respectively (Fig. 5J and K). Strain AM-5 and AM-8 also improved germination parameters under alkali stress. Alkali stress caused significantly greater damage to control seeds than salt stress across all measured parameters.

### Effects of bacterial inoculation on wheat seedling growth and physiology under saline-alkali stress

**Fig. 6:**
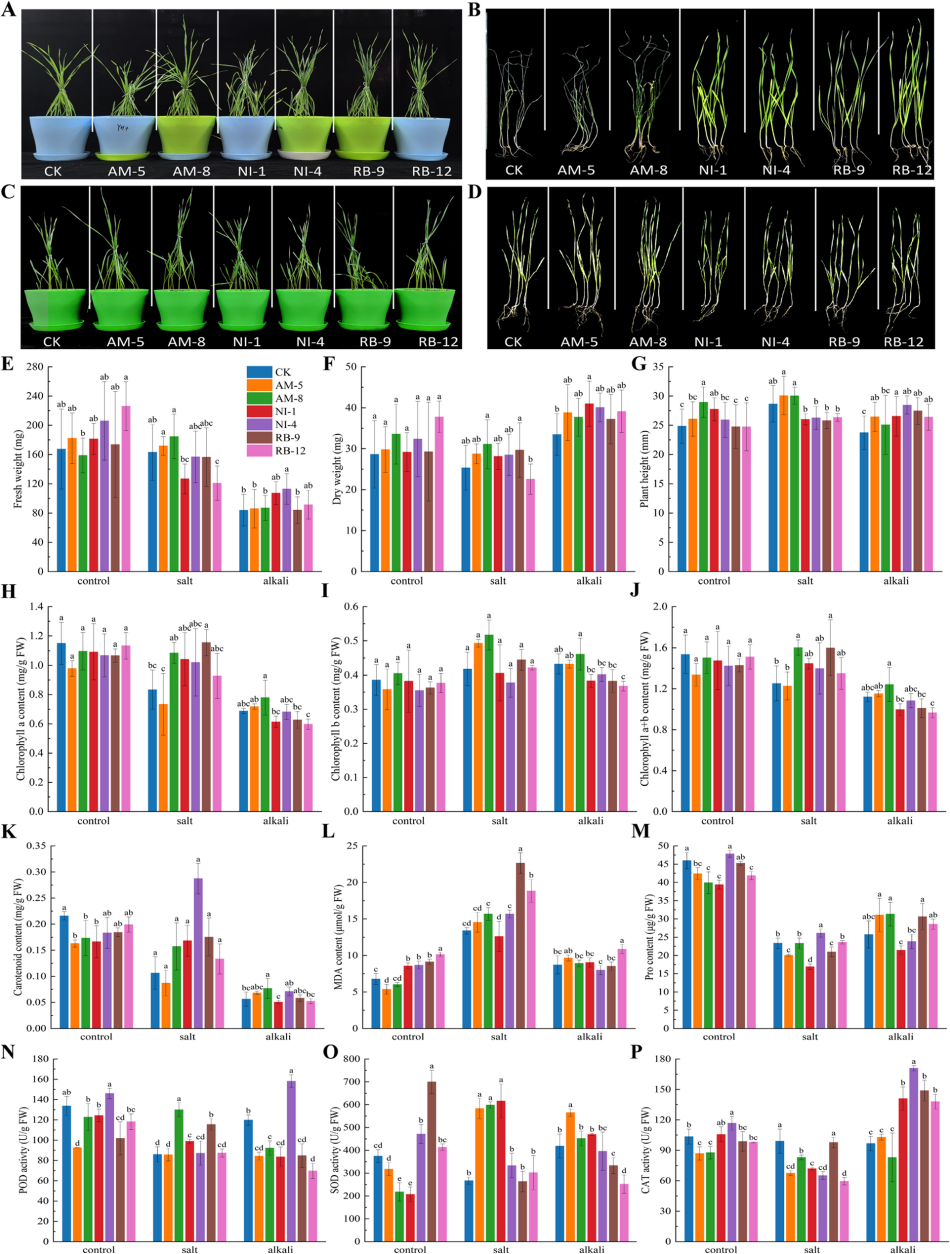
Effects of six selected endophytic bacterial strains on growth and physiological parameters of wheat seedlings under normal, salt, and alkali stress. **A**, **C** Representative images of wheat seedlings at 30 days under a normal and c salt/alkali stress conditions. **B**, **D** Separate panels of the seedling images shown in (A) and (C), respectively. **E** Fresh weight, **F** dry weight, **G** plant height, **H** chlorophyll a content, **I** chlorophyll b content, **J** total chlorophyll content, **K** carotenoid content, **L** malondialdehyde (MDA) content, **M** proline content, **N** peroxidase (POD) activity, **O** superoxide dismutase (SOD) activity, and **P** catalase (CAT) activity. Data are means ± SE (*n* = 3). Asterisks indicate significant differences compared to the respective non-inoculated control (*p < 0.05, **p < 0.01, ***p < 0.001)

Bacterial inoculation significantly influenced wheat seedling growth and stress physiology. Under normal conditions, inoculated seedlings showed increased fresh weight (FW), dry weight (DW), and plant height compared to controls, with strains AM-8, NI-4, and RB-12 showing particularly positive effects (Fig. 6E and G).

Under salt stress, all inoculated seedlings had higher DW than controls. Strain AM-8 demonstrated comprehensive mitigation, enhancing DW, FW, and plant height by 22.85%, 13.23%, and 4.67%, respectively. Under alkali stress, NI-1 and NI-4 were superior, increasing plant height, FW, and DW by up to 19.54%, 34.85%, and 22.34%, respectively (Fig. 6E and G). Inoculated plants often exhibited darker leaves and more robust root systems (Fig. 6A and D).

Photosynthetic pigment content responded differently to stress and inoculation. Salt stress, combined with inoculation by strains like AM-8 and NI-4, led to significant increases in chlorophyll a (Chla), chlorophyll b (Chlb), and carotenoid (Car) content compared to stressed controls (Fig. 6H and K). Alkali stress severely reduced pigments in controls, an effect partially ameliorated by some bacterial treatments.

Oxidative stress markers and antioxidant responses were strain- and stress-specific. MDA levels increased under salt stress but were reduced by strains AM-5 and AM-8 under normal conditions (Fig. 6L). Proline accumulation was induced differentially; for example, NI-4 increased it under salt stress, while AM-5/AM-8 did so under alkali stress (Fig. 6M). Antioxidant enzyme activities (POD, SOD, CAT) were modulated in a complex manner, with AM-8 showing synergistic activation of SOD and POD under salt stress (Fig. 6N and P).

## Discussion

This study provides a systematic characterization of the seed endophytic bacterial communities in ten Dao-di medicinal plants from Gansu Province. By integrating high-throughput sequencing with cultivation-based techniques, we move beyond descriptive cataloging to offer insights into the assembly rules, potential functions, and application prospects of these microbiomes. Our data robustly confirm the paramount influence of host genotype in structuring the seed bacterial community, a pattern consistent across plant kingdoms [16, 20]. However, the significance of our findings lies in extending this general principle to the ecologically and economically crucial context of Dao-di herbs, revealing how host specificity manifests and its potential implications for medicinal plant biology and cultivation.

The observed phylogenetic conservation at the phylum level (dominance of Proteobacteria, Firmicutes, etc.) aligns with global seed microbiome studies [18, 38], suggesting fundamental constraints of the seed environment. The striking divergence at the genus level, however, is more informative. The enrichment of *Sphingomonas* in legumes versus *Pseudomonas* in NI points to a fine-tuned selection process likely mediated by host-specific seed exudates, surface compounds, or internal immune responses [39]. This is not merely taxonomic trivia; it suggests that the host plant actively recruits or filters microbes based on traits beneficial to its specific ecological strategy or secondary metabolism. The existence of a substantial core microbiome (219 genera) across phylogenetically diverse hosts is intriguing. This core may represent vertically transmitted, essential mutualists or resilient environmental taxa commonly found in Gansu’s agricultural soils. Its functional role warrants targeted investigation, as it could underpin basic seed physiology common to these medicinal species.

A key finding is the dramatically reduced diversity in NI, as indicated by Shannon and Simpson indices. This is not an artifact but a likely biological signal of intense host selective pressure, possibly driven by potent antimicrobial compounds characteristic of this medicinal species. While a low-diversity microbiome might suggest specialization and efficient host control, it also raises an important practical challenge: such a simplified microbial community could limit the plant’s resilience to biotic and abiotic stresses, potentially making it more vulnerable in changing environments or monoculture settings. This duality between specialization and fragility is a crucial point for sustainable cultivation strategies.

The functional predictions, while acknowledging the limitations of PICRUSt2, suggest a compelling functional stratification alongside taxonomic divergence. The association of NI’s microbiome with environmental adaptation genes, HP with genetic information processing, and *Glycyrrhiza* with nutrient metabolism hints at differentiated microbial contributions to host fitness. For instance, the unique phyla identified in HP [40–43] strongly imply a microbiome adapted to local stress factors like salinity or heavy metals, directly linking community composition to geo-authenticity (Dao-di) pressures.

The stark contrast between sequencing and cultivation results—with cultivable isolates dominated by *Bacillus* and *Pseudomonas*—vividly illustrates the “great plate count anomaly” and the vast uncultured majority [44, 45]. This is not just a methodological footnote but a central challenge for application. The promising plant-growth-promoting (PGP) traits (IAA production, nitrogen fixation, phosphate solubilization) found in the cultivable fraction, particularly in strains like *Bacillus amyloliquefaciens* [46–48], *Pantoea*, and *Pseudomonas* [49, 50], provide a tangible starting point for developing bio-inoculants [51, 52]. However, the translation gap from in vitro PGP activity to consistent field efficacy in medicinal plants is substantial. Future work must address formulation, delivery, and ecological competition to ensure these strains can successfully integrate into the established, host-specific seed microbiome.

In conclusion, this study elucidates the highly customized nature of seed microbiomes in Dao-di herbs, governed by host selection. We propose that these microbial communities are not passive passengers but active contributors to host adaptation, possibly influencing aspects of medicinal quality and stress resilience. The immediate application lies in using these host-specific microbial signatures as novel indicators for assessing seed quality or origin. For long-term impact, the isolated PGP strains offer potential for microbiome-assisted precision agriculture. The critical next steps must involve mechanistic studies to unravel the molecular dialogue (e.g., via seed exudate profiling) that shapes this assembly, metabolomic correlations to link key microbes to medicinal compound biosynthesis, and rigorous field trials to validate the agronomic benefits of microbial inoculants. Furthermore, although synthetic community (SynCom) experiments were beyond the scope of the present study, the construction of SynComs that integrate complementary PGP traits represents a logical and powerful future direction to enhance functional robustness, ecological resilience, and host adaptation under complex field conditions. Establishing a dedicated culture collection for Dao-di medicinal plant microbes will be essential to drive this transition from observational science to sustainable innovation.

## Conclusion

This study demonstrates that seeds of ten medicinal plants from Gansu harbor host-specific endophytic bacterial communities with a small shared core microbiome. A cultivable subset, predominantly *Bacillus* and *Pseudomonas*, displayed multiple in vitro plant growth-promoting traits. Inoculation with selected strains enhanced wheat germination and alleviated saline-alkaline stress in proof-of-concept assays. These findings establish medicinal plant seeds as reservoirs of functionally promising bacteria and highlight host identity as a key determinant for bioprospecting. However, direct application potential remains contingent on validation using the original host species. This work provides a foundational dataset and culture collection to support future seed microbiome research in medicinal plants.

## Supplementary Information

Supplementary Material 1.

## Data Availability

The raw sequencing reads generated in this study have been deposited in the Zenodo repository and are publicly available under the accession DOI: https://doi.org/10.5281/zenodo.15855276.
