# Supplementary material for: Study on the diversity, structure, and function of endophytic bacteria in seeds of genuine medicinal plants in gansu province
Source: BMC Plant Biol. 2026 Apr 18;26:936. doi: 10.1186/s12870-026-08767-5 (PMC13224594; doi:10.1186/s12870-026-08767-5)
Supplement: Supplementary file 1 — Supplementary Material 1. [file 12870_2026_8767_MOESM1_ESM.docx]

**Supplementary Material**

**Study on the Diversity, Structure, and Function of Endophytic Bacteria in Seeds of Genuine Medicinal Plants in Gansu Province**


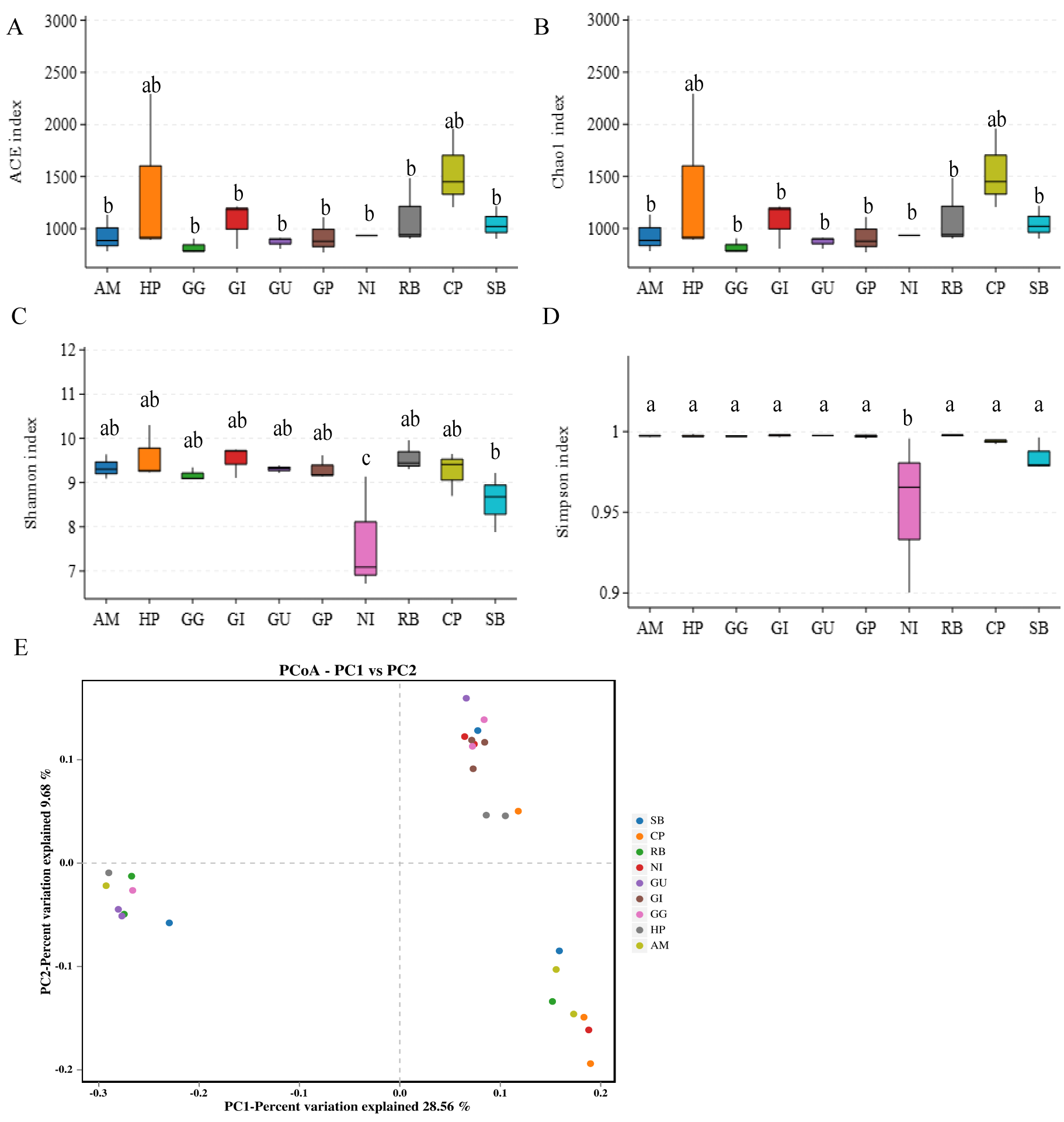


**Figure S1**. Alpha- and beta-diversity of seed endophytic bacterial communities. ****A**** ACE index. ****B**** Chao1 index. ****C**** Shannon index. ****D**** Simpson index. Alpha diversity (including Shannon index for species diversity, Chao1 index for richness, and Pielou’s evenness index) was evaluated, and intergroup differences were compared using the Kruskal–Wallis test. ****E**** Principal coordinates analysis (PCoA) based on Bray–Curtis distances, with group differences tested by PERMANOVA (999 permutations). Different lowercase letters above bars or near symbols indicate statistically significant differences between seed groups (*p*<0.05).


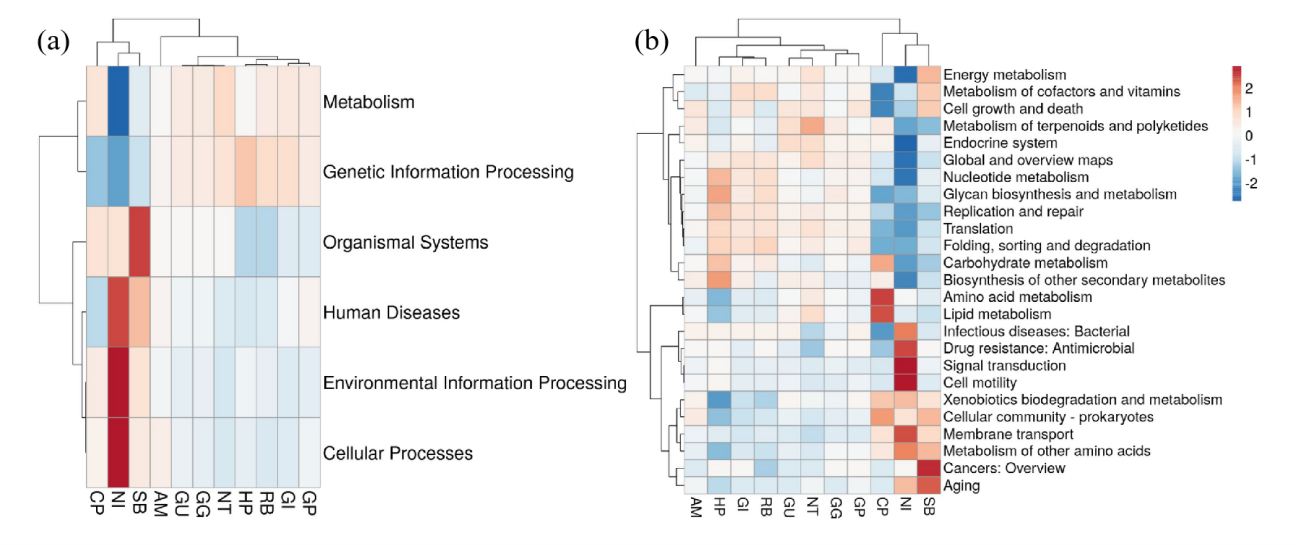


**Figure S2**: Functional prediction of seed endophytic bacterial communities based on KEGG pathways. **A** Relative abundance of predicted KEGG pathways at level 1. **B** Heatmap of predicted KEGG pathways at level 2 showing functional variation among seed communities.


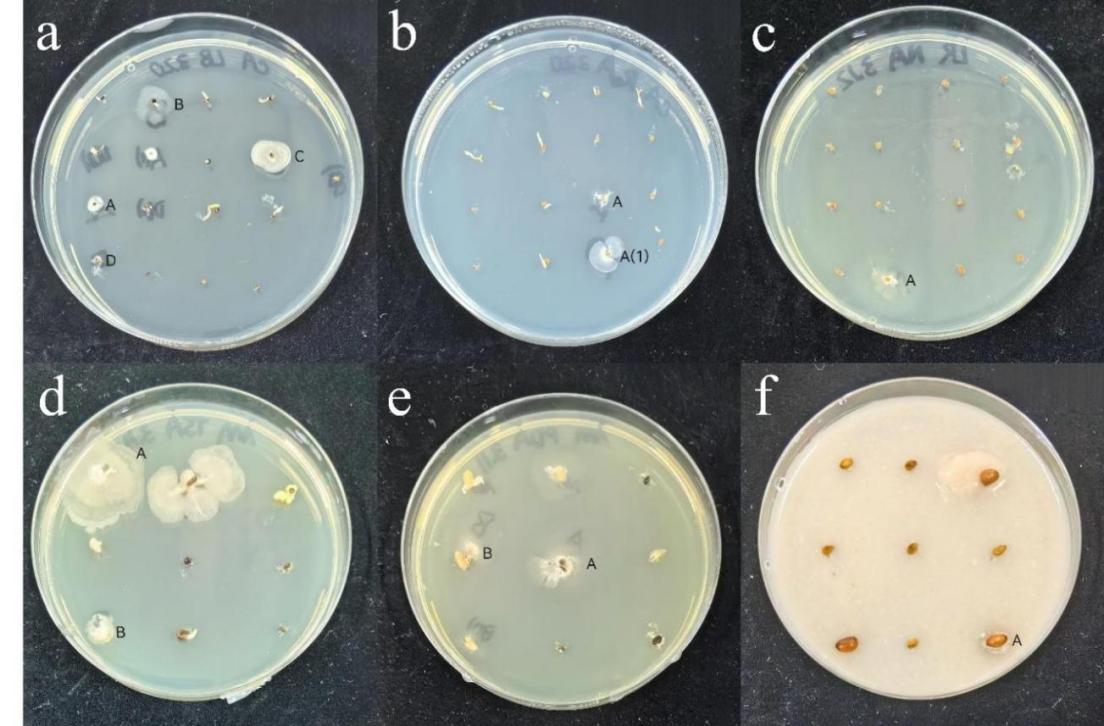


**Figure S3.** Colonial morphology of endophytic bacteria isolated from seeds. All images show results from isolation experiments using the sterile seed embedding method. The culture media used are as follows: a: LB agar medium, b: R2A agar medium, c: Nutrient Agar (NA) medium, d: Tryptic Soy Agar (TSA) medium, e: Potato Dextrose Agar (PDA) medium, f: Oatmeal Agar (OA) medium.


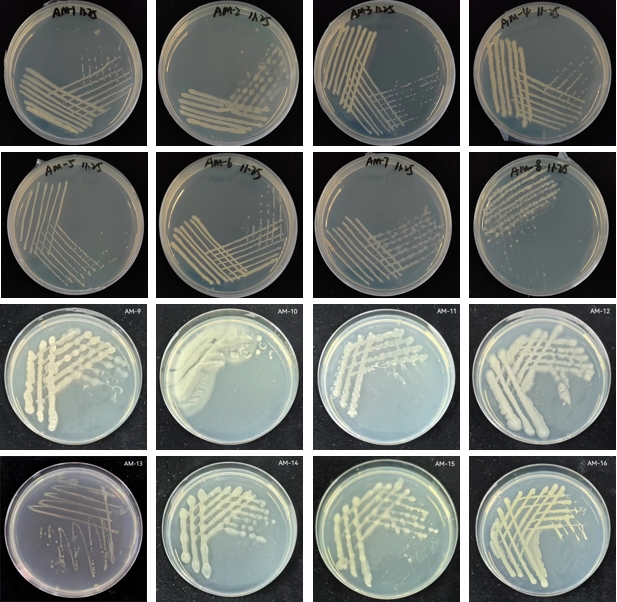


**Figure S4** Endophytic bacteria isolated from AM seeds, arranged in order from

AM-1 to AM-16.


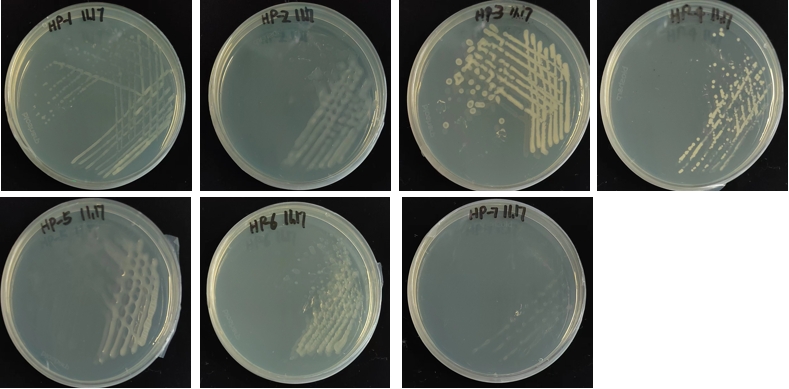


**Figure S5** Endophytic bacteria isolated from HP seeds, arranged in order from

HP-1 to HP-7.


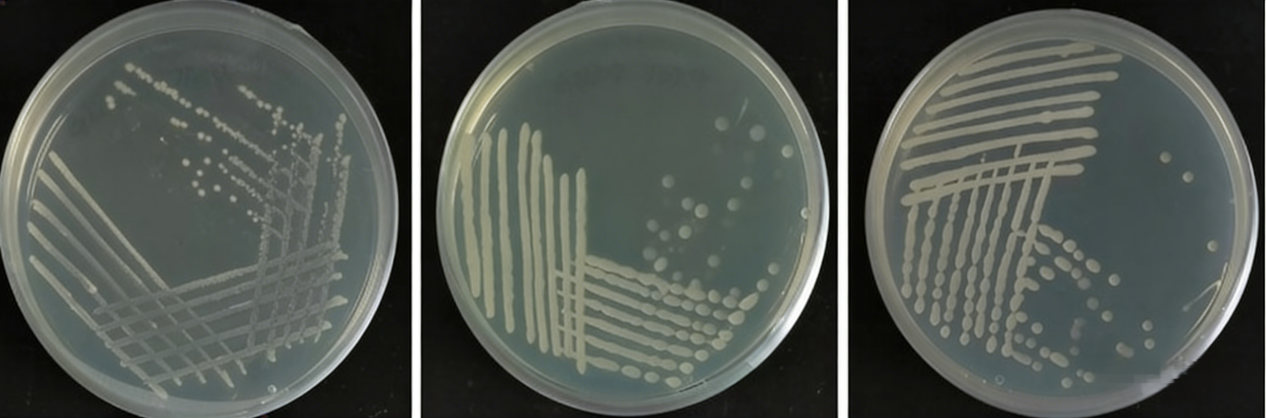


**Figure S6** Endophytic bacteria isolated from GG seeds, arranged in order from

GG-1 to GG-3.


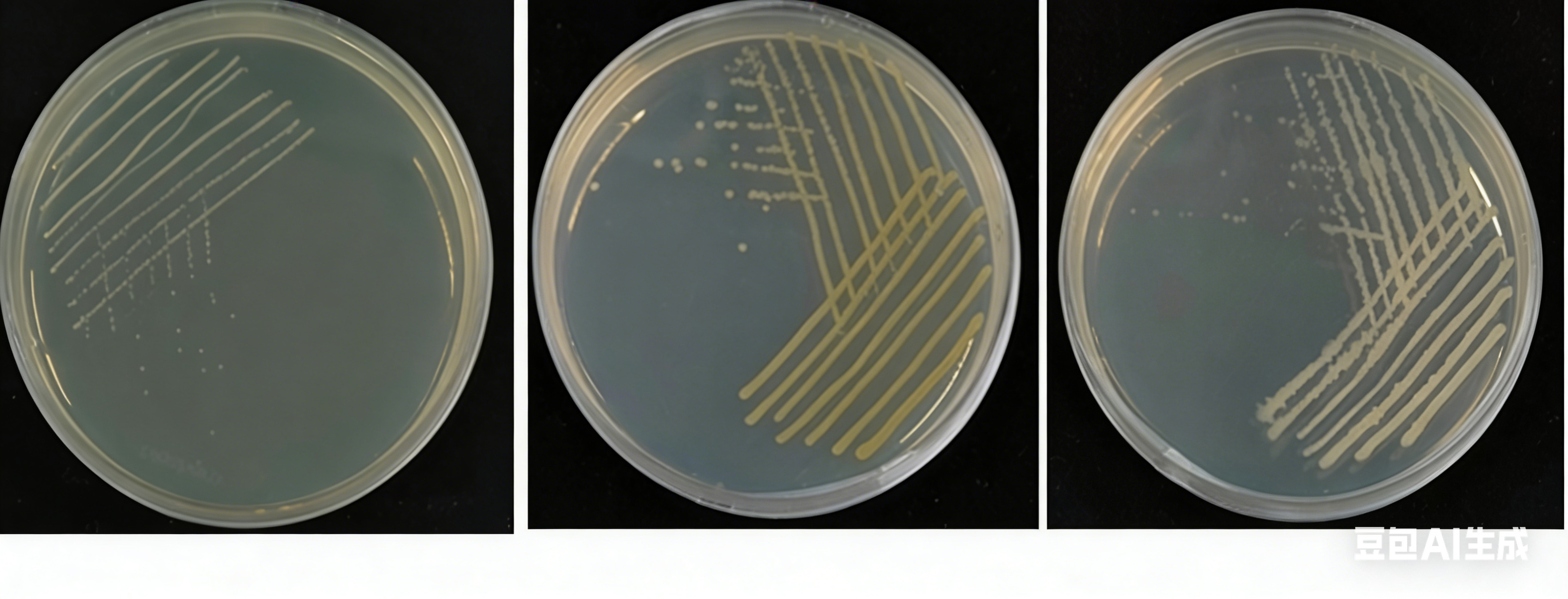


**Figure S7** Endophytic bacteria isolated from GI seeds, arranged in order from

GI-1 to GI-3.


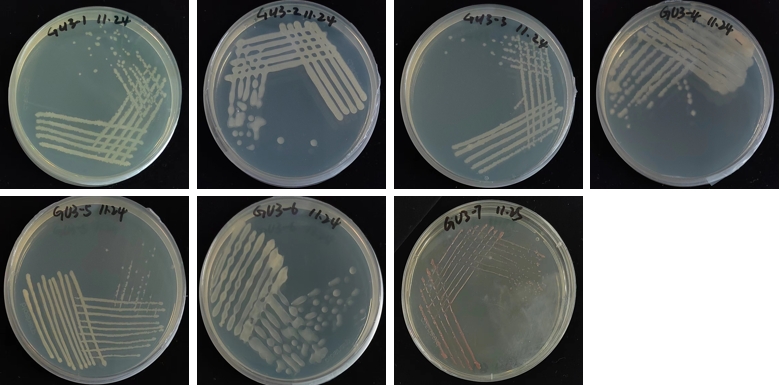


**Figure S8** Endophytic bacteria isolated from GU seeds, arranged in order from

GU-1 to GU-7.


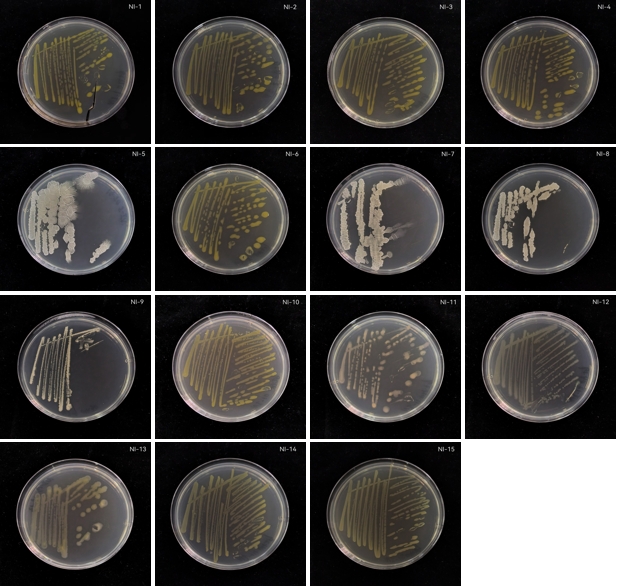


**Figure S9** Endophytic bacteria isolated from NI seeds, arranged in order from

NI-1 to NI-15.


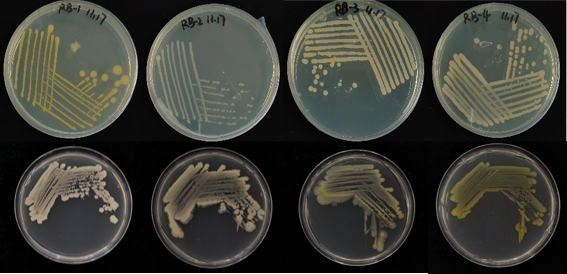


**Figure S10** Endophytic bacteria isolated from RB seeds, arranged in order from

RB-1 to RB-8.


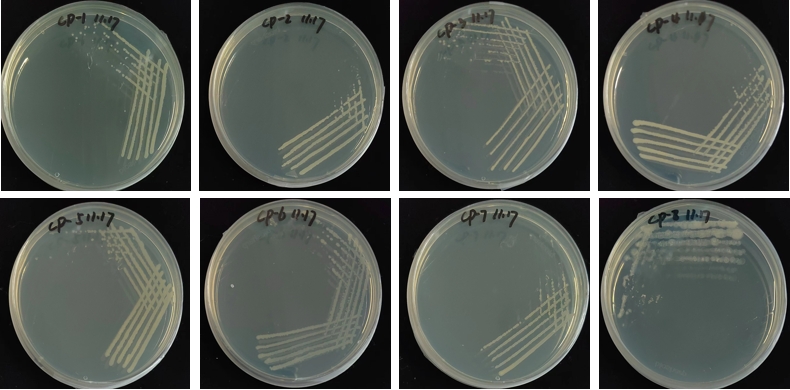


**Figure S11** Endophytic bacteria isolated from CP seeds, arranged in order from

CP-1 to CP-8.


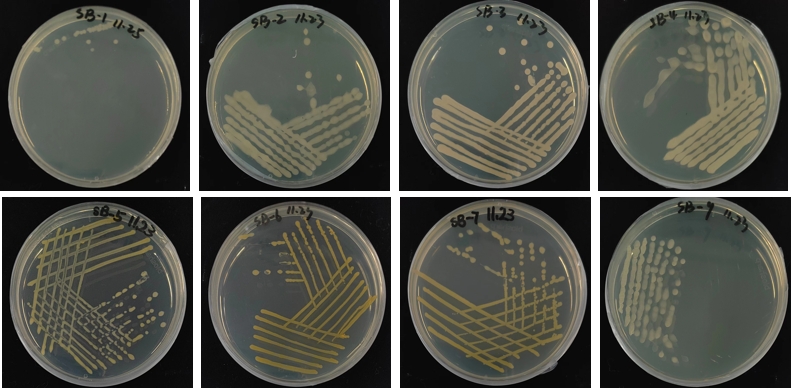


**Figure S 12** Endophytic bacteria isolated from SB seeds, arranged in order from

SB-1 to SB-7 and SB-9.

****Table S1. Identification of endophytic bacteria isolated from AM seeds****

| Phylum | Genus | Species | Strain Count | Strain ID | Similar strain name in NCBI Database | Reference  numbers  (NCBI) | Similarity  (%) |
| --- | --- | --- | --- | --- | --- | --- | --- |
| Bacillota | Bacillus | B. pumilus | 2 | AM-1 | Bacillus pumilus  CIP 52.67 | NR_115334.1 | 98.18% |
|  |  |  |  | AM-4 | Bacillus pumilus  NBRC 12092 | NR_112637.1 | 98.98% |
|  |  | B. stercoris | 1 | AM-2 | Bacillus stercoris  D7XPN1 | NR_181952.1 | 97.15% |
|  |  | B. zhangzhouensis | 1 | AM-3 | Bacillus zhangzhouensis  MCCC 1A08372 | NR_148786.1 | 97.49% |
|  |  | B. paralicheniformis | 1 | AM-7 | Bacillus paralicheniformis  KJ-16 | NR_137421.1 | 99.44% |
|  |  | B. safensis | 1 | AM-6 | Bacillus safensis  NBRC 100820 | NR_113945.1 | 99.23% |
|  |  | B. tequilensis | 1 | AM-8 | Bacillus tequilensis 10b | NR_104919.1 | 97.66% |
|  |  | B. subtilis | 4 | AM-9 | Bacillus subtilis S58 | OQ504806.1 | 98.92% |
|  |  |  |  | AM-13 | Bacillus subtilis  IAM 12118 | NR_112116.2 | 96.89% |
|  |  |  |  | AM-14 | Bacillus subtilis N-11 | MW345828.1 | 99.53% |
|  |  |  |  | AM-15 | Bacillus subtilis T21 | OP984801.1 | 99.44% |
|  |  | B. aerius | 2 | AM-10 | Bacillus aerius 24K | NR_118439.1 | 97.60% |
|  |  |  |  | AM-16 | Bacillus aerius 24K | NR_118439.1 | 98.03% |
|  |  | B. licheniformis | 1 | AM-11 | Bacillus licheniformis  DSM 13 | NR_118996.1 | 90.81% |
|  |  | B. halotolerans | 1 | AM-12 | Bacillus halotolerans  ATCC 25096 | OQ876681.1 | 99.63% |
| Pseudomonadota | Prolinoborus | P. fasciculus | 1 | AM-5 | Prolinoborus fasciculus  CIP 103579 | NR_104948.1 | 99.32% |

****Table S2. Identification of endophytic bacteria isolated from HP seeds****

| Phylum | Genus | Species | Strain Count | Strain ID | Similar strain name in NCBI Database | Reference  numbers  (NCBI) | Similarity  (%) |
| --- | --- | --- | --- | --- | --- | --- | --- |
| Bacillota | Bacillus | B. subtilis | 3 | HP-1 | Bacillus subtilis Md1-37 | MF581443.1 | 100.00% |
|  |  |  |  | HP-6 | Bacillus subtilis T22 | OP984802.1 | 99.81% |
|  |  |  |  | HP-7 | Bacillus subtilis IIILI-9 | MK367793.1 | 99.61% |
|  |  | B. stercoris | 1 | HP-2 | Bacillus stercoris D7XPN1 | NR_181952.1 | 99.59% |
|  |  | B. tequilensis | 1 | HP-5 | Bacillus tequilensis G06 | OQ405609.1 | 99.81% |
|  | Priestia | P. megaterium | 2 | HP-3 | Priestia megaterium B21 | MG709189.1 | 99.90% |
|  |  |  |  | HP-4 | Priestia megaterium NBRC 15308 = ATCC 14581 | NR_112636.1 | 99.61% |

****Table S3. Identification of endophytic bacteria isolated from GG seeds****

| Phylum | Genus | Species | Strain Count | Strain ID | Similar strain name in NCBI Database | Reference  numbers  (NCBI) | Similarity  (%) |
| --- | --- | --- | --- | --- | --- | --- | --- |
| Bacillota | Paenibacillus | P. illinoisensis | 1 | GG-1 | Paenibacillus illinoisensis  NBRC 15959 | NR_113828.1 | 98.24% |
|  | Bacillus | B. safensis | 1 | GG-3 | Bacillus safensis FO-36b | NR_041794.1 | 99.93% |
|  |  | B. pumilus | 1 | GG-4 | Bacillus pumilus CIP 52.67 | NR_115334.1 | 99.48% |
| Pseudomonadota | Pantoea | P. agglomerans | 1 | GG-2 | Pantoea agglomerans  DSM 3493 | NR_041978.1 | 97.52% |

****Table S4. Identification of endophytic bacteria isolated from GI seeds****

| Phylum | Genus | Species | Strain Count | Strain ID | Similar strain name in NCBI Database | Reference  numbers  (NCBI) | Similarity  (%) |
| --- | --- | --- | --- | --- | --- | --- | --- |
| Bacillota | Paenibacillus | P. polymyxa | 1 | GI-1 | Paenibacillus polymyxa  NBRC 15309 | NR_112641.1 | 97.84% |
|  | Bacillus | B. halotolerans | 2 | GI-2 | Bacillus halotolerans  LMG 22477 | NR_115931.1 | 98.53% |
|  |  |  |  | GI-3 | Bacillus halotolerans  CECT 5687 | NR_115930.1 | 98.56% |

****Table S5. Identification of endophytic bacteria isolated from GU seeds****

| Phylum | Genus | Species | Strain Count | Strain ID | Similar strain name in NCBI Database | Reference  numbers  (NCBI) | Similarity  (%) |
| --- | --- | --- | --- | --- | --- | --- | --- |
| Bacillota | Bacillus | B. velezensis | 1 | GU-1 | Bacillus velezensis  FZB42 | NR_075005.2 | 98.55% |
|  |  | B. mojavensis | 2 | GU-2 | Bacillus mojavensis  NBRC 15718 | NR_112725.1 | 99.16% |
|  |  |  |  | GU-4 | Bacillus mojavensis  IFO 15718 | NR_024693.1 | 99.14% |

****Table S6. Identification of endophytic bacteria isolated from NI seeds****

| Phylum | Genus | Species | Strain Count | Strain ID | Similar strain name in NCBI Database | Reference  numbers  (NCBI) | Similarity  (%) |
| --- | --- | --- | --- | --- | --- | --- | --- |
| Bacillota | Bacillus | B. subtilis | 2 | NI-5 | Bacillus subtilis sp.168 | NR_102783.2 | 98.09% |
|  |  |  |  | NI-8 | Bacillus subtilis  NRRL NRS-744 | NR_116192.1 | 99.26% |
|  |  | B. stercoris | 1 | NI-7 | Bacillus stercoris D7XPN1 | NR_181952.1 | 99.34% |
|  |  | B. mojavensis | 1 | NI-6 | Bacillus mojavensis  NBRC 15718 | NR_112725.1 | 99.44% |
|  | Paenibacillus | P. xylanivorans | 1 | NI-9 | Paenibacillus xylanivorans A59 | NR_178867.1 | 98.61% |
|  |  | P. chibensis | 1 | NI-11 | Paenibacillus chibensis 4R1 | NR_178813.1 | 98.37% |
| Pseudomonadota | Pantoea  Pantoea | P. agglomerans | 5 | NI-1 | Pantoea agglomerans DSM 3493 | NR_041978.1 | 98.86% |
|  |  |  |  | NI-2 | Pantoea agglomerans NCTC9381 | NR_114735.1 | 98.74% |
| Pseudomonadota |  |  |  | NI-4 | Pantoea agglomerans ATCC 27155 | NR_114505.1 | 99.12% |
|  |  |  |  | NI-14 | Pantoea agglomerans  LMG 1286 | NR_116751.1 | 90.95% |
|  |  |  |  | NI-15 | Pantoea agglomerans  NBRC 102470 | NR_114111.1 | 98.39% |
|  |  | P. vagans | 1 | NI-3 | Pantoea vagans  LMG 24199 | NR_116115.1 | 99.03% |
|  |  | P. brenneri | 1 | NI-10 | Pantoea brenneri  LMG 5343 | NR_116748.1 | 98.00% |
|  | Pseudomonas | P. viridiflava | 2 | NI-12 | Pseudomonas viridiflava  ATCC 13223 | NR_114482.1 | 99.17% |
|  |  |  |  | NI-13 | Pseudomonas viridiflava | NR_117825.1 | 99.44% |

****Table S7. Identification of endophytic bacteria isolated from RB seeds****

| Phylum | Genus | Species | Strain Count | Strain ID | Similar strain name in NCBI Database | Reference  numbers  (NCBI) | Similarity  (%) |
| --- | --- | --- | --- | --- | --- | --- | --- |
| Bacillota | Bacillus | B. stercoris | 1 | RB-2 | Bacillus stercoris  D7XPN1 | NR_181952.1 | 98.25% |
|  | PeriBacillus | P. simplex | 2 | RB-3 | PeriBacillus simplex  NBRC 15720 = DSM 1321 | NR_112726.1 | 98.66% |
|  |  |  |  | RB-4 | PeriBacillus simplex  LMG 11160 | NR_114919.1 | 99.27% |
| Pseudomonadota | Pantoea | P. brenneri | 1 | RB-1 | Pantoea brenneri  LMG 5343 | NR_116748.1 | 97.76% |
|  |  | P. agglomerans | 1 | RB-9 | Pantoea agglomerans  DSM 3493 | NR_041978.1 | 97.27% |

****Table S8. Identification of endophytic bacteria isolated from CP seeds****

| Phylum | Genus | Species | Strain Count | Strain ID | Similar strain name in NCBI Database | Reference  numbers  (NCBI) | Similarity  (%) |
| --- | --- | --- | --- | --- | --- | --- | --- |
| Bacillota | Bacillus | B. altitudinis | 2 | CP-1 | Bacillus altitudinis LXJ71 | MN746195.1 | 99.80% |
|  |  |  |  | CP-2 | Bacillus altitudinis 41KF2b | NR_042337.1 | 99.80% |
|  |  | B. zhangzhouensis | 1 | CP-4 | Bacillus zhangzhouensis  MCCC 1A08372 | NR_148786.1 | 99.61% |
|  |  | B. pumilus | 1 | CP-5 | Bacillus pumilus CIP 52.67 | NR_115334.1 | 99.61% |
|  |  | B. cabrialesii | 1 | CP-6 | Bacillus cabrialesii TE3 | NR_180419.1 | 99.60% |

****Table S9. Identification of endophytic bacteria isolated from SB seeds****

| Phylum | Genus | Species | Strain Count | Strain ID | Similar strain name in NCBI Database | Reference  numbers  (NCBI) | Similarity  (%) |
| --- | --- | --- | --- | --- | --- | --- | --- |
| Bacillota | Bacillus | B. subtilis | 3 | SB-1 | Bacillus subtilis BCRC 10255 | NR_116017.1 | 99.29% |
|  |  |  |  | SB-8 | Bacillus subtilis DSM 10 | NR_027552.1 | 99.51% |
|  |  |  |  | SB-9 | Bacillus subtilis IAM 12118 | NR_112116.2 | 99.61% |
|  |  | B. stercoris | 1 | SB-2 | Bacillus stercoris D7XPN1 | NR_181952.1 | 99.60% |
|  |  | B. tequilensis | 1 | SB-4 | Bacillus tequilensis 10b | NR_104919.1 | 99.58% |
|  | PeriBacillus | P. simplex | 1 | SB-3 | PeriBacillus simplex  NBRC 15720 | NR_112726.1 | 99.41% |
| Pseudomonadota | Erwinia | E. persicina | 1 | SB-5 | Erwinia persicina  NBRC 102418 | NR_114078.1 | 98.21% |
|  | Pantoea | P. agglomerans | 2 | SB-6 | Pantoea agglomerans  DSM 3493 | NR_041978.1 | 99.60% |
|  |  |  |  | SB-7 | Pantoea agglomerans  NCTC9381 | NR_114735.1 | 99.22% |
